# FceRI density and spontaneous secretion from human basophils

**DOI:** 10.1371/journal.pone.0179734

**Published:** 2017-07-03

**Authors:** Donald MacGlashan

**Affiliations:** Johns Hopkins Asthma and Allergy Center, Baltimore, MD; Cornell University, UNITED STATES

## Abstract

Mast cells and basophils depend on aggregation of the high-affinity IgE receptor, FceRI, to initiate secretion. A variety of studies have shown that FceRI densities vary 100 fold among subjects’ basophils and it has been speculated that high densities might be responsible for unusual behaviors of the cells, notably sensitivity to certain monomeric IgE antibodies or spontaneous release. These studies experimentally examined the density dependence of spontaneous release and signaling element expression in subjects’ basophils with FceRI densities ranging from approximately 6000 to 600,000 per cell. Through the use of numerical simulation, this study examined the expectations for spontaneous receptor aggregation and aggregate persistence at densities of FceRI ranging from 5000 to 500,000. Experimentally, FceRI density was not associated with greater spontaneous histamine release even when secretion was enhanced by the inclusion of deuterium oxide in the buffers. There was also no association of 15 activating or de-activating signaling elements with FceRI density. The numerical simulations demonstrated that at densities of ≈500,000 receptors, 13% of receptors were involved in transient aggregates at any given moment but that these aggregates rarely persisted for greater than 10 milliseconds. In contrast, a weak linear antigen aggregator, with ligand affinities known to induce secretion, would generate aggregates persisting for an average of ≈60 milliseconds. These results suggest that although a high density of FceRI likely produces a large number of transient aggregates, these aggregates do not persist long enough to induce signaling that results in secretion and do not induce the cells to alter their expression of several signaling elements known to be important in regulating secretion from human basophils. The results set some boundaries on the aggregation requirements for inducing histamine release from human basophils.

## Introduction

Allergic reactions result from the IgE-mediated (immunoglobulin E) activation of mast cells and basophils. These cells express high affinity receptor for IgE (FceRI) and bound IgE confers on these cells the ability to sense the antigenic environment of the cells. A remarkable feature of FceRI expression on basophils and mast cells is the range of possible expression. After synthesis and placement on the cell surface, the residency time of the unoccupied receptor is relatively short; 50% are returned to the inside of the cell every 24 hours. Some of these might be recycled to the cell surface but many are degraded. However, as near as can be determined, if FceRI binds IgE, it will never leave the cell surface, provided aggregation doesn’t occur. This relationship between IgE and the stability of expressed FceRI produces some remarkable levels of FceRI expression. The steady state density of FceRI in the absence of IgE is approximately 5000–10000 per cell (a number which is dependent on the synthetic rate) [1]. With very high IgE concentrations (> 50 µg/ml), FceRI densities have been observed as high as 1 million per cell [2]. The average atopic subject has basophils expressing 250,000 per cell [3]. It is not difficult to imagine that signal transduction pathways have to adapt to the extreme range of surface expression. The specific reason is that this is a receptor that depends on aggregation to initiate signaling.

**Fig 1:**
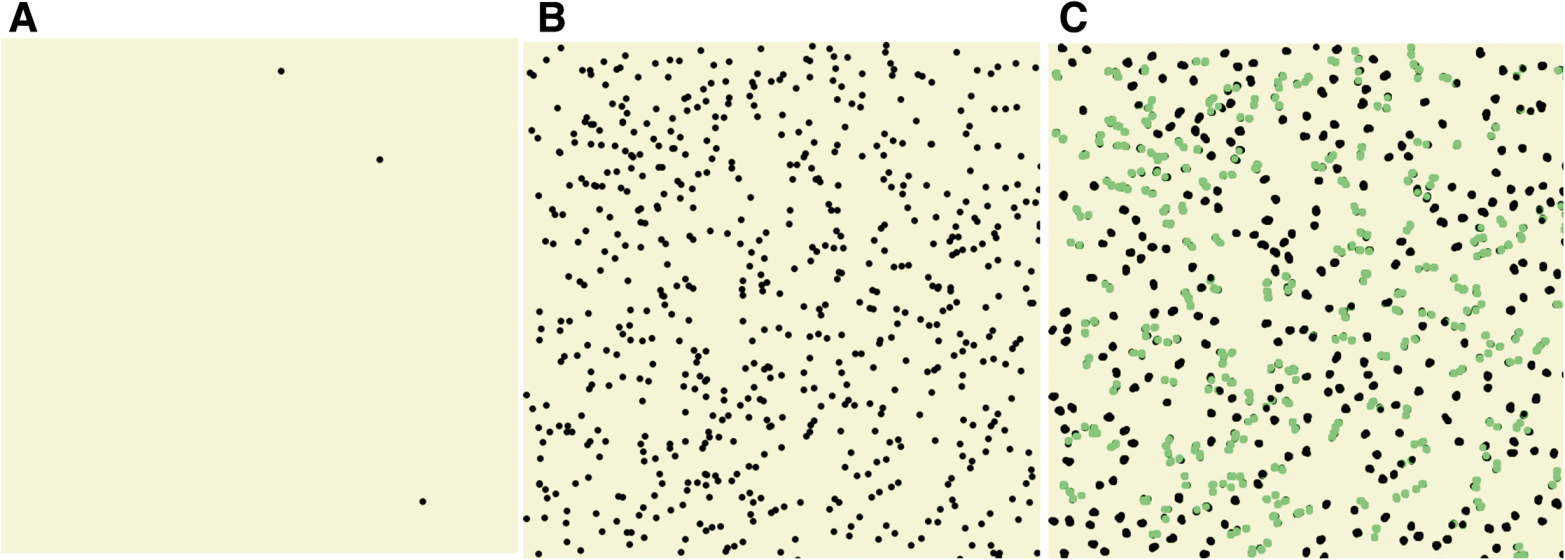
Representation of an “over-head” view of receptor density and associations. Each “dot” represents the radius of gyration of a surface IgE molecule. Panel A; density of 5000/cell. Panel B; density of 500,000/cell. Panel C; density of 500,000/cell where green dots represent IgE-FceRI molecules in close enough proximity to be considered a potentially functional aggregate based on published studies.

Fig 1 shows what the surface of a hypothetical cell would look like solely from the perspective of the IgE bound FceRI. The radius of gyration for the IgE molecules is approximately 65 angstroms [4]. With a simple circle to represent the sweep of the IgE molecule as it rotates around its center of mass, Fig 1 uses the small black circles to represent this sweep radius for bound IgE. The image represents a 1 µm “square” on the surface of the idealized spherical cell. The reality is considerably more complicated since cells have numerous folds increasing the surface area of the membrane but also having restricted zones through which receptor-bound IgE does not traverse [5]. So the idealized sphere is a rough approximation of the accessible membrane of a 10 µm diameter cell. Fig 1A shows a density of 5000 per cell while Fig 1B shows a density of 500,000 per cell. What is immediately evident with a random placement of IgE/receptor at this latter density is that there are many spontaneous aggregates in this simple static image (Fig 1C, green circles). When this image is set in motion, assuming Brownian motion for the receptor moving in the plane of the plasma membrane, it becomes obvious that random associations are frequent but transient. The question is whether these transient associations are capable of inducing meaningful signaling and how many are required to matter.

Regarding whether the spontaneous aggregates might matter, there are a variety of experimental results that provide some context for the question. Another property of FceRI biology is that while some form of aggregation is required to initiate signaling, it doesn’t have to be a physical crosslink [6–8]. Simply increasing the local density of the receptor is sufficient to imbalance what appears to be a finely tuned balance of phosphorylation and de-phosphorylation reactions that are constantly present on the inner membrane near the receptor [9, 10]. In addition to not requiring physical crosslinks, the distance between nearby receptors can be quite large [11]. Some studies have suggested that 200 nm is still considered “adjacent” from the perspective of the signaling steps that follow. In contrast, there are also indications that stronger signals result from closer proximity [12]. These kinds of results have implications for the question about spontaneous aggregation.

The question of transience is a harder to answer but two experimental observations provide some insight. First, the signaling cascade actually has differential sensitivity to the transience of the aggregate depending on the readout of activation being assessed. Notably, extremely weak crosslinkers can still drive a signaling reaction that results in gene transcription while not leading to degranulation [13]. A second set of observations relates to studies in which bivalent haptens were used to initiate a crosslinking reaction. For human basophils, the cells were sensitized with a penicillin (BPO)-specific IgE and stimulated with bivalent penicillin (two benzyl-penicilloyl groups linked by a 6 or 8 carbon chain aliphatic molecule). This stimulus generates linear chains of aggregates although the evidence suggests that simple dimeric aggregates are the predominant size formed [14–16]. Calculations based on the affinity of IgE for the BPO moiety suggest that a singly bound BPO2 to a BPO-specific IgE molecule has a T1/2 for persistence of 70 msec and when both BPO ends of BPO2 are involved in a crosslink, the linkage breaks with a time constant of approximately 300 msec [4, 15]. That said, it is not explicitly known what duration is necessary for a functional crosslink but BPO2 does induce histamine release and for the basophils of some subjects, histamine release is marked. In some individuals, fewer than 50 BPO-specific IgE cell surface Abs that form long-lived stable complex aggregates (e.g., with a multivalent BPO-HSA (human serum albumin) as the antigen) are sufficient to drive histamine release [17] while for a stimulus like BPO2, 500–1000 are required [18]. But since basophils may have hundreds of thousands of receptors, this represents a small fraction of what could be available. Addition of monovalent BPO-FLYS (BPO coupled to formyl-lysine) to an ongoing BPO2-initiated reaction stops the reaction as rapidly as adding EDTA [15] to starve the reaction of extracellular calcium needed for the activation signal. The BPO2 reaction also re-equilibrates rapidly, within seconds, even without the addition of BPO-FLYS [15]. These observations suggest that the duration of the aggregate need not be very long.

Therefore, if the juxtaposition of FceRI molecules need not be very long or close, the question becomes, how dense do the receptors need to be to initiate spontaneous signaling? This issue may have been inadvertently addressed in studies purporting to show that monomeric IgE can induce secretion [19]. Many subsequent studies have explored this unexpected observation. The bottom-line is that there are some IgE molecules that when bound to FceRI, induce signaling while others do not [20, 21]. The current thinking is that this happens with higher FceRI densities and possibly by a weak (or strong as in the case of the monoclonal murine IgE, SPE7) interaction of the IgE to each other or other cell surface molecules [21, 22]. A theoretical treatment of the problem by Pecht et al [23] explored the fraction of receptors that might be considered spontaneously aggregated as a function of surface density.

Methodology for answering the question of transience experimentally has not been developed. An alternative approach, an aggregation simulation model, was used to explore the potential for long duration spontaneous aggregates.

## Experimental methods

### Subjects & ethics

Leukapheresis donors are approved by protocols obtained by the leukapheresis center and the cells used for purification of basophils are considered a residual waste product. The protocol for obtaining blood from the subjects that were not processed with leukapheresis was approved by the Johns Hopkins IRB and the donors provided a written approval of a consent approved by the same IRB. This specific study was encompassed by the above approvals. Written consents are saved as part of the standard record keeping for IRB-approved studies.

### Materials

The following were purchased: PIPES ((piperazine-N,N-bis-2-ethanesulfonic acid), bovine serum albumin (BSA), EGTA, EDTA, D-glucose, NaF, Na4P2O7, Na3VO4, 2-ME, NP-40, FMLP (Sigma, St. Louis, MO); crystallized human serum albumin (HSA) (Miles Laboratories, Elkhart, IN); fetal calf serum (FCS) and RPMI 1640 containing 25 mM HEPES and L-glutamine (BioWhittaker, Walkersville, MD); Percoll, (Pharmacia, Piscataway, NJ); Goat anti-human IgE Ab was prepared as previously described [17].

### Buffers

PIPES-albumin-glucose (PAG) buffer consisted of 25 mM PIPES, 110 mM NaCl, 5 mM KCl, 0.1% glucose, and 0.003% HSA. PAGCM was PAG supplemented with 1 mM CaCl2 and 1 mM MgCl2. PAG-EDTA consisted of PAG supplemented with 4 mM EDTA (ethylenediamine N, N, N’, N’- tetraacetic acid).

### Histamine release reaction

Blood was partially enriched for basophils using single-step Percoll gradients. After washing to obtain a mononuclear cell preparation, cells were resuspended PAGCM buffer (to assess histamine release). Total histamine content was obtained by lysis of the cell preparation with 1.6% perchloric acid. These experiments included two types of spontaneous release assessments. The standard measurement is in PAGCM buffer without stimulus but a second measurement included 44% deuterium oxide to replace the water in the PAGCM. D2O (deuterium oxide) is known to enhance secretion of any type of stimulation [24]. Histamine release was calculated as (stimulated release–spontaneous release)/total histamine content.

### Histamine measurements

Histamine was measured by automated fluorimetry [25]. The percentage of total histamine release was calculated after subtraction of spontaneous histamine release.

### Basophil cell surface IgE measurement

On a separate occasion, basophils from the same subjects were analyzed for cell surface total IgE using acetate elution. An aliquot of cells was used to determine the total basophil count by alcian blue staining and the remaining cells were treated with acetate buffer, pH 3.7, to dissociate endogenous IgE. The recovered supernatant was neutralized to pH 7 and IgE concentration measured by a total IgE assay (immunoCap). A calculation of the number of IgE molecules present and the cell number allowed the cell surface IgE density to be determined [26]. As described previously [27], calibration of the antibody (22E7) used to detect FceRI by flow cytometry involved a comparison of flow cytometric results with explicit measurement of IgE eluted from a counted number of basophils. The flow cytometric results were then equated with this measured density of IgE/basophil.

### Basophil purification

Basophils were purified from leukapheresis packs or from blood obtained by venipuncture. When used at high purity, they were purified to near homogeneity by sequential application of Percoll gradients, countercurrent elutriation and negative selection using the basophil purification kit (Stem Cell Technologies, Vancouver, BC) and columns from Miltenyi Biotec (Aubum, CA) [28]. The average purity of these basophils by alcian blue staining [29] was 99%. Starting viability of these cells was typically >97%.

### Calibrated Western blots

These methods and a presentation of example blots from these studies have been previously published [30]. All sampling was based on lysing equivalent cell numbers as this approach removed the assumption that cells from different subjects would contain either equivalent amounts of total protein or equivalent amounts of a reference protein (e.g., actin; normalizing to protein is traditional but not appropriate when there is no a priori knowledge about the assumption of equivalence). The purified basophils were counted and 300,000 pelleted cells were lysed in 20 µl of hot ESB, the tube placed in boiling water for 5 minutes and the samples stored at -80˚C until electrophoresis. When 6–8 samples were accumulated, they were run in a 15 well 8% tris-glycine gel with molecular weight markers in the first and last lanes. After blocking, the nitrocellulose was cut horizontally at the 98 kD marker level. The >98 kD and <98 kD portions were blotted on two or three successive days with a choice of antibodies that did not interfere with each other. No stripping step occurred between blotting. Many of these antibodies produced single bands at the loading levels in this study so that they do not interfere with each other. In addition, in the successive blotting, the antibodies were chosen to be progressively more sensitive detectors so there is little evident cross-over of information between measurements. Each Western blot included a three point standard curve of a basophil lysate prepared prior to the survey (aliquoted and stored at –80˚C) to bracket the range of expected expression for each of the elements tested. Multiple film exposures were made in order to optimize linearity, the films were digitized and the band intensities calculated against the standard curve [30].

## Simulation methods

To make predictions about the number and duration of spontaneous aggregates, a simulation of IgE/receptor associations with or without physical linkers was developed. The supporting information contains a detailed description of the methodology as appendix 1 and the relevant code framework file (S3 File) for the simulation.

## Experimental results

**Fig 2:**
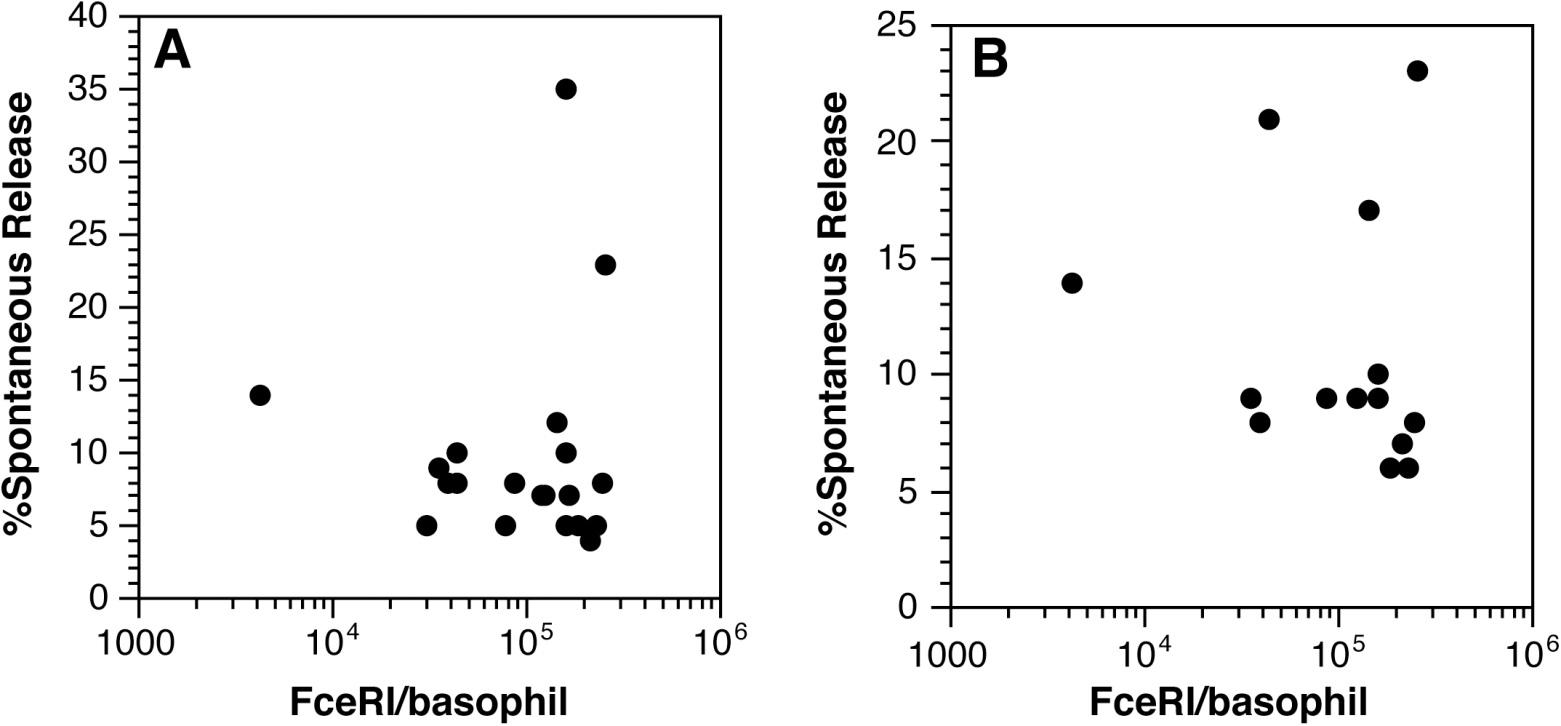
Correlations between FceRI density and in vitro spontaneous histamine release from human basophils. Panel A, normal buffer; Panel B, D2O in buffer. (Some experiments in this survey did not include D2O).

It is reasonable to suggest that the resting mechanisms of regulation of secretion may need to track with the receptor density in order to not allow spontaneous, non antigen-induced, secretion. A starting point for determining if receptor expression has an impact on function was to determine whether there was spontaneous secretion that was dependent on the density of FceRI. This was done in 4 separate surveys. In two of these surveys, spontaneous secretion was assessed in normal buffers and in buffer replacing 44% of the water with deuterium oxide. This substitution is known to significantly augment secretion, even without stimulation [31]. Fig 2 shows one of the surveys to illustrate the results. Table 1 summarizes the correlations for the 4 surveys. With one exception, there were no statistically significant correlations. The inverse correlation observed in survey 2 was puzzling since samples from the same experiment stimulated in D2O did not show a correlation. Taking into account that 5 of the 6 correlations were not significant, it seems likely that there is no relationship between FceRI density and spontaneous release.

**Table 1:** Summary of correlations for FceRI density vs. spontaneous release.

	R	n	p
Survey 1	0.03^a^	20	0.89
Survey 1 (D2O)	-0.17	14	0.55
Survey 2	-0.59	32	0.0004
Survey 2 (D2O)	-024	32	0.18
Survey 3	-0.28	30	0.14
Survey 4	0.22	13	0.48

^a^See S2 File for the data that led to these statistics.

**Fig 3:**
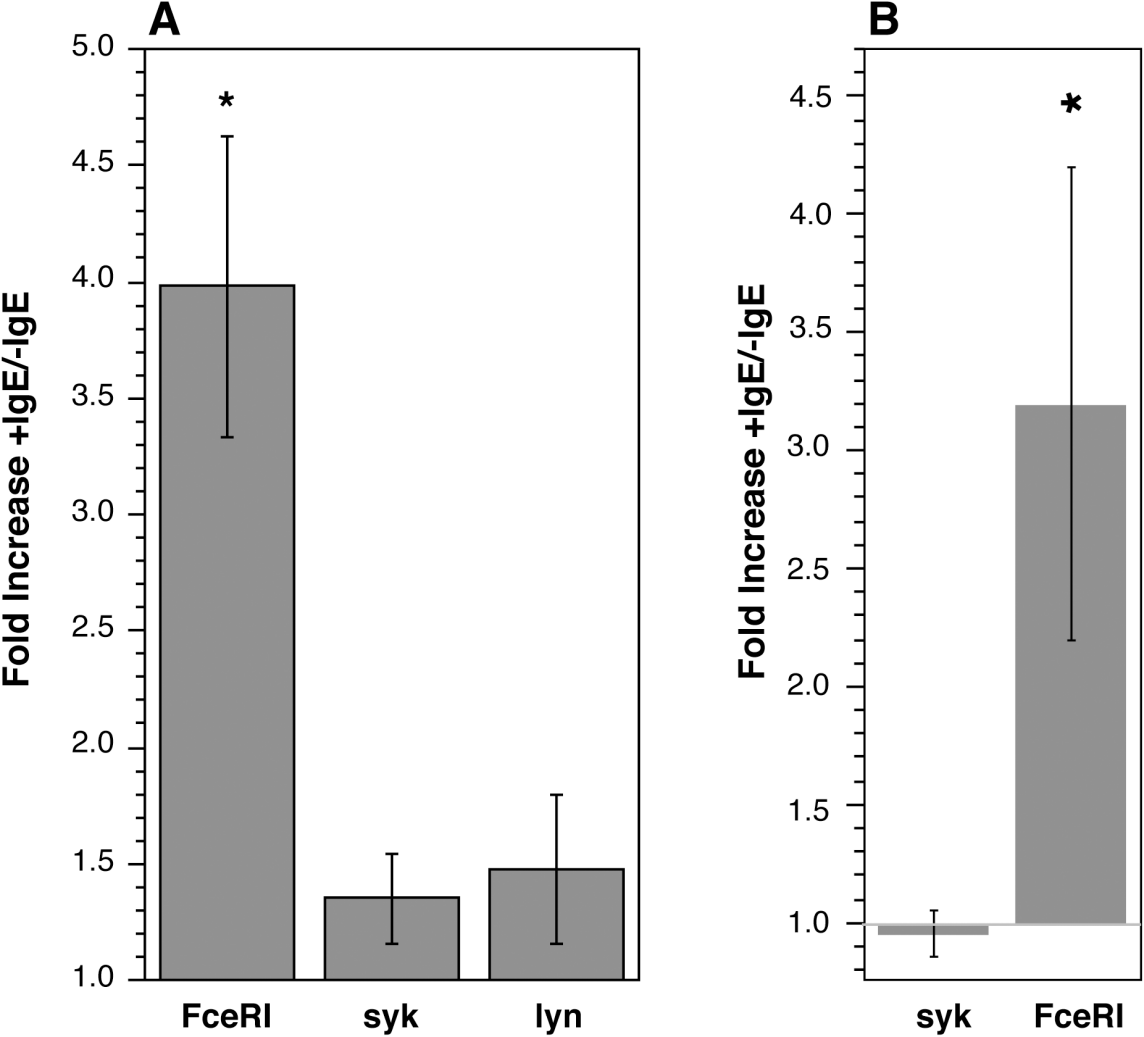
Changes in FceRI, syk or lyn expression during up-regulation of FceRI by culture for 4 days in 5 ng/ml IL-3 ± 1 µg/ml IgE. Panel A, peripheral blood basophils (n = 3). Panel B, CD34B starting on day 17 of culture (n = 4).

If spontaneous secretion is not dependent on receptor density, there may be changes in signaling elements that maintain a quiescent state. A first look at this issue was whether peripheral blood basophils (PBB) cultured in IL-3 or CD34-derived basophils (CD34B) cultured in IL-3 and assessed for changes in signaling species show changes related to changes in FceRI. Two kinases that can be shown to increase the signaling and secretion of basophils are the src-family kinase, lyn, and syk [32]. However, despite a 4 fold increase in receptor (induced by culture with IgE), neither lyn nor syk increase in cultured PBB or CD34B (Fig 3).

A survey of the natural distribution of several signaling elements in subjects’ basophils across a spectrum of IgE densities did not reveal marked differences at the high and low end of the density spectrum for FceRI. Table 2 shows the results for a range of 35,000 to 572,000 receptors (n = 7) for syk and Table 3 summarizes the results for other signaling molecules (see also the supporting information for detailed information for each of the elements shown in Table 3). The results for PLCg1 were suggestive of a relationship so this particular species was re-examined in a separate survey of 8 subjects with a receptor range of 8600 to 124000 receptors per basophil. In addition to PLCg1, the negative regulator cbp/PAG was examined; neither signaling element showed a correlation with receptor density (PLCg1: R = -0.48, p = 0.24, for cbp/PAG: R = -55, p = 0.15).

**Table 2:** A single example of FceRI expression vs. syk expression (in units of Western blot standard).

FceRI	log(FceRI)	Syk
35000	4.54	0.444
40000	4.60	0.954
44000	4.64	0.101
65000	4.81	0.276
150000	5.18	0.360
165000	5.22	0.076
572000	5.76	0.219

**Table 3:** Pearson R for each element vs. FceRI; all p>0.05.

Element	N	R
syk^a^	7	-0.302
Lyn	7	0.522
p85a	7	0.387
Fyn	6	0.510
p110d	6	-0.145
btk	6	-0.071
PLCg2	6	-0.006
PLCg1	5	-0.819
SOS2	5	-0.474
BOB1	5	-0.510
CIN85	5	-0.666
SHIP1	7	0.270
SHIP2	7	0.530
c-cbl	7	0.182
PTEN	5	-0.298
SHP-1	5	-0.195

^a^See S3 File for the data that led to these statistics.

It is also possible to derive a small amount of data from recent studies examining the change in FceRI expression during treatment of patients with omalizumab. Only 4 signaling species have been examined in peripheral blood basophils, syk, lyn, c-cbl, and cbp/PAG (the syk and c-cbl data have been reported previously [33], indeed, the syk results have been observed in multiple studies [26, 34]). With a reduction in receptor to 16% of pre-treatment levels, there were no changes in c-cbl [33], lyn, or cbp/PAG (the latter two were 1.05±0.07 and 1.06±0.08 of pre-challenge levels) but there was a average 2-fold increase in syk [33].

## Simulation results

To provide a context for the lack of a relationship between receptor density and spontaneous secretion, a simulation of the generation of spontaneous aggregates was performed. For comparative purposes, a related simulation examined the persistence of clusters forming in a reaction with a small bivalent hapten, a ligand that has been demonstrated to induce secretion. The supporting information describes the results of the simulation in detail. The general conclusion is that while very large numbers of spontaneous aggregates form at high receptor densities—at 270,000 receptors/cell, 10–20% of the receptors can be involved in an cluster (see S1 File, and S1 Fig)–the average lifetime of such clusters is much shorter (no lifetimes greater than 15 msec and most < 2 msec) than clusters that form during a crosslinking reaction (> 50 msec, see S6 Fig) even if the crosslinker has relatively fast dissociation constants as modeled in the simulation.

## Discussion

These studies were motivated by the observations in murine mast cells that a monomeric-bound IgE could induce secretion. Although this observation is largely restricted to murine mast cells sensitized with only a few specific murine IgE antibodies (so-called cytokinergic Abs), an interesting issue was raised regarding the impact of high densities of FceRI on spontaneous activation of basophils or mast cells. With a 100-fold range of FceRI expression, it would be surprising if the cells didn’t accommodate the signaling apparatus for conditions that might lead to a great deal of spontaneous aggregation. One can only speculate on the precise reason basophils and mast cells accumulate so many FceRI. One plausible explanation is to prevent a parasite from inducing nonspecific IgE as a defense against specific IgE-mediated secretion as part of the host rejection mechanism. Whatever the reason, the large up-regulation presents a potential problem for the secretory apparatus.

The simulations demonstrate that at the highest densities of FceRI that are observed in individuals with high IgE levels, there are a considerable number of spontaneous aggregates present. As noted previously, a variety of studies have suggested that juxtaposition need not be close [11] but it does appear that some stability of the aggregate is required for a full secretory reaction [35]. Short-lived aggregates may nevertheless induce partial cellular responses, notably the transcriptional regulation of cytokine or chemokine secretion [13]. The simulation suggests that aggregates that persist for greater than 10 milliseconds are rare even at high densities and in this context, even a weak crosslinker performs much better, producing aggregates that persist for 50–100 milliseconds. Since published studies show that these weak crosslinkers, e.g, BPO2, or DNP2, induce secretion [14, 17, 36] while cells that express high densities of FceRI do not show spontaneous release that is related to FceRI density (this paper’s results), we conclude that for secretion to occur, aggregates must persist for times exceeding 10 milliseconds.

The experimental findings show that there was no relationship between FceRI density and spontaneous histamine release ± deuterium oxide. In other studies we have shown that there is also no relationship between cellular sensitivity (the number of IgE molecules needed for a half-maximal response) or maximum histamine release [2]. In other words, there is no indication that the high densities shift the balance of reactions in any particular direction.

The observation that there is no relationship between FceRI density and spontaneous release might suggest that the cell uses counter-balancing mechanisms to keep activation in check. One way to counter-balance the increase in receptor is to down-regulate activating signaling steps. Alternatively, up-regulation of termination mechanisms could suppress spontaneous activation. We found no evidence for either of these changes in the molecular species that have been studied in basophils and shown to be participatory during IgE-mediated activation and de-activation. However, the list is short and there remain many other signaling species that would need examination.

In addition, there is no a priori reason that any single signaling element might show a correlation with FceRI density. It may be that the entire signal element network adjusts such that only small increments of change occur for any one element. With this in mind, a simple calculation was performed on the data set used for the results in Table 2. After normalizing all results (each level of expression was recast as a fractional standard deviation from the mean for a particular element), all known activating elements were summed and from this was subtracted the sum of all known terminating elements (the reasoning being that an inverse correlation would occur for positive elements and positive correlation for negative elements) and the synthesized aggregate of all these elements compared with the receptor density. This was done for 15 elements and the result was a correlation of 0.86 (p = 0.045, n = 5 donors) was obtained against receptor expression (or R = 0.83 against log[receptor], p = 0.065). Strictly speaking, this one model was only one of 2^30 possible models for 15 signaling elements and if this manipulation were generalized then the p-value that would be considered significant would need to be Bonferonni adjusted by 2^30 and this clearly will never result in a statistically significant result when n = 5–7. Nevertheless, a general answer to the question of whether signaling elements adjust their expression according to receptor density cannot escape the need to consider the entire relevant network of elements.

In a limited pilot study of 2 patients treated with omalizumab and 2 treated with placebo, there were no notable changes in the transciptome of basophils that experienced both down-regulated FceRI during treatment and up-regulated syk expression relative to the placebo-treated group (unpublished results). Notably, there were no changes in mRNA expression for a wide variety of expressed signaling species. What is notable from studies of omalizumab treatment is that omalizumab does down-regulate FceRI expression while up-regulating syk expression [33]. This would suggest a link between FceRI density and syk. However, this linkage is likely distinct from what we are searching for in these studies. The reason is that down-regulation of FceRI always occurs, and to similar extents during treatment but up-regulation of syk does not, only occurring if syk expression was low prior to treatment. This observation de-links the two events from the kind of regulation strictly related to receptor changes. Despite the dramatic change in receptor expression, there is little beyond syk expression that has been observed to change in the signaling apparatus. Three other relevant species have been examined, lyn,cbp/PAG, and c-cbl [33] and one of these change during omalizumab-mediated down-regulation of FceRI.

Even though the persistence of spontaneous aggregates is short, there are approximately 100,000 of them at any given moment on basophils with 500,000 receptors. It remains somewhat surprising that there is no notable consequence to this situation. Perhaps the fact that FceRI has no intrinsic enzymatic activity allows the system to behave this way [9]. It would be easy to imagine that if it did have kinase activity, e.g., like EGF receptor, that density swings this wide would need to invoke a change in the signaling apparatus. But if the proximal kinases, such as lyn, fyn or syk, do not change their expression levels while FceRI increases or decreases, perhaps this acts a buffer for these changes. The chemical reaction would still be driven more strongly towards early activation events but without significant persistence, and perhaps with little hysteresis in returning to the “resting” state by termination processes, activation is blunted sufficiently. Further study of the entire signaling apparatus would be needed to draw stronger conclusions about the processes involved.

## Supporting information

S1 File Appendix text (PDF). Description of the aggregation simulation.
(PDF)

S2 File Data used to generate Table 1. Excel spreadsheet.
(XLSX)

S3 File Data used to generate Table 2. Excel spreadsheet.
(XLSX)

S4 File Code for simulation. Standard C code; main and model code without user interface code. To relate line numbers in the appendix to the code in this file, a code editor is needed.
(C) Click here for additional data file.

S1 Fig Functions called at each time step of the simulation. (TIF)

S2 Fig Determining aggregate persistence. Each IgE molecule was tracked for its association with an aggregate and marked for size of the aggregate at each cycle. A record for all IgEs at each cycle was stored and later scanned for each IgE and its status with respect to aggregate size. If it was marked as in an aggregate of size ≥2 (the gray zone in Fig 3), then a counter was started. Once returning to size 1, the counter was stopped and the length of time tallied. The figures shows one particular IgE for a period of only 15 cycles (750 µsec) and it would count 2 events (1 only 50 µsec in length and one 300 µsec in length and passing briefly through an aggregate of size 4) and the start of a third event cut short in this plot.
(TIF)

S3 Fig Spontaneous aggregation size. Panel A; results of simulation for a 13.5 (filled circles) and 20.0 (filled squares) nm center-to-center separation distance being counted as an aggregate without regard to size or persistence at a given moment. Panel B; relationship between the size of the spontaneous aggregate, the number of aggregates and density of FceRI. The numbers on the ordinate reflect the total number of aggregates (to calculate the total number of IgE/receptor involved, multiply by the aggregate size (e.g., a pentamer aggregate = 5 total receptors involved).
(TIF)

S4 Fig Spontaneous aggregation persistence. Persistence for clusters/aggregates of any size larger than monomers for two center-to-center separation distances (circle = 13.5 nm, square = 20.0 nm). The count is for the number of such clusters for the first 75 msec of the simulation at a total receptor density of 270,000.
(TIF)

S5 Fig Concentration-dependence for IgE crosslink formation using a bivalent crosslinker as determined by the simulation. A single-site affinity of 5x10^6^ M^-1^ was used for the simulation.
(TIF)

S6 Fig Comparison of persistence times for spontaneous clusters vs. bivalent ligand-induced crosslinks for a weak crosslinker that is known to induce histamine release. Panel A, 3 simulations, all at 270,000 receptors/cell, spontaneous aggregates (open squares), an optimal concentration (10^−7^ M) of crosslinker (black circles), or suboptimal crosslinker (2.5x10^-10^ M) (green open diamonds). The histograms on the right edge of the plot show the remaining number of aggregates at times > 75 msecs. Panel B, 3 simulations, at two different densities of receptor; spontaneous aggregates at 270,000 receptors/cell (open squares), an optimal concentration (10^−7^ M) of crosslinker at 270,000 receptors/cell (black circles), or optimal crosslinker (10^−7^ M) at 13,500 receptors/cell (green open circles). The histograms on the right edge of the plot show the remaining number of aggregates at times > 75 msecs. Panel C; 2 simulations, kr (dissociation constant) = 10 (, as in panel A) or = 100 (green squares) both at 270,000 receptors/cell.
(TIF)
