# Supplementary material for: FceRI density and spontaneous secretion from human basophils
Source: PLoS One. 2017 Jul 3;12(7):e0179734. doi: 10.1371/journal.pone.0179734 (PMC5495218; doi:10.1371/journal.pone.0179734)
Supplement: S1 File — Description of the aggregation simulation. (PDF) [file pone.0179734.s001.pdf]

Appendix: Simulation Methods

To make predictions about the number and duration of spontaneous aggregates, a simulation of IgE/receptor associations with or without physical linkers was developed.

Underlying principles of the model

The cell surface was simplified to a flat plane but a plane whose edges wrap to the opposite edge (functionally, a Euclidean 2-torus). This topology should adequately mimic a spherical shell without worrying about the calculations using spherical coordinates. Stochastic motion, binding and crosslinking formed the basis for simulating aggregation states. A time-step for incrementing the global state of receptor/IgE position and linkage was chosen to be small enough to capture a range of possible probabilities of ligand binding and receptor motion but large enough to allow the simulation to run efficiently. Ligand itself was not explicitly included in the model, its presence was simulated by a stochastic calculation at each time-step that would set a Boolean field in the data structure that represented surface IgE that one Fab arm was occupied or not. In effect, ligand is not depleted during the reaction, a situation that is frequently the case in experimental studies.

Each IgE/receptor was programmatically represented by a data structure that had 6 data fields, two fields representing each IgE arm's bound state and 2 fields representing which IgE (of all the possible IgE data structures) an arm was bound to (2 additional fields were used for internal bookkeeping during the simulation). An

additional data structure (indexing of which was the same as the IgE data structure) represented the position in 2D space for a particular IgE. Whether ligand-based linking was being studied or spontaneous association of mobile receptors, there were data structures that created linked lists of aggregated IgE data structures. If the simulation was only monitoring spontaneous cluster formation, the stochastic aspect of ligand binding was not used unless there was a test for increased association between IgE/receptor (see below). There was a variant R-tree data structure used to keep track of any formed clusters that allowed the program to dynamically track cluster size and cluster duration.

To improve the simulation rate, multiple processors were used (see code line 580 in File_S4). "Edges" were part of the simulated surface, so the software kept track of edge issues, allowing the wrapping of motion across edge boundaries. An internal clock triggers a routine that performs the needed calculations at each time-step which are shown in S1 fig. (see code line 423 in the S4_File.c in the supplement) As noted above, the simulation's time-step is fixed, and its duration was chosen to encompass the range of possible probabilities for motion and ligand binding based on real world constants for ligand binding and dissociation, crosslinking constants and Brownian motion of the IgE or IgE clusters. The time-step was chosen to be 50 microseconds. In this period of time a Markov-like probability of transition between states was pre-calculated for each type of transition. Tests for similar results at different timesteps (e.g., distance traveled for a short simulation was 1.45 ± 0.76, 1.47 ± 0.77, 1.45 ±0.76, 1.44 ± 0.75 model units for timesteps of 100, 50, 25, 12.5

µsec) and, in particular, convergence to the analytically predicted distance traveled for a period of time showed that the diffusion simulation was accurate.

There were three random number generators used for all calculations, either generating a yes-no bit (random bit generator), a float value ranging from 0 to 1 (uniform deviate generator), or a Gaussian random z-number generator (producing a random fractional deviation from the mean) [1]. These software random number generators were seeded with values that were drawn from an internal clock (in order to initiate each simulation with different random sequences). A new seed based on information from outside the simulation was used every several tens-of-thousands of visits to the software generators. This was done to minimize random number bias across many uses of the generators. The random number generator used was described in 'Numerical Recipes in C' [1]. Prior to the start of the simulation, all the various time constants for the binding reactions were re-cast as step probabilities. This includes the linking probabilities once two IgE molecules were calculated to be within a critical distant of each other [2, 3].

1- Update Positions

The motion of IgE was stochastic and for simplicity, when IgE was linked to other IgEs, the cluster/aggregate moved as one entity (code line 919 or 3887 of S4_File)). In other words, at each time step, each IgE position was updated but if the IgE were part of a cluster/aggregate, the first calculated positional change in the cluster was propagated throughout the IgE molecules within the cluster and no further movements were calculated for the remaining IgE molecules within the cluster.

Motion was calculated according to the diffusion equation for proteins within the membrane but like other simulated steps, the actual calculation is based on a pre-calculated probability distribution for diffusion within the time-step interval (using published values for FceRI diffusion).

To reduce calls to the Gaussian random generator, a single call to the Gaussian generator was made to obtain the distance moved in 2 dimensions followed by a single call to the uniform deviate generator to pick a direction (angle). With these two calls, the distance moved in x and y directions was calculated. Fick diffusion in 2 dimensions is Gaussian with the 2-dimensional sigma for the distribution:

$$\text{“sigma”} = \text{sqrt}(4 * D_T * \text{timestep}) \quad (1)$$

where D_T is the translational diffusion constant [3]; $D_T = 2x10^{-10}$ cm^2/sec. In this case, a single pick from the Gaussian random number generator function generates a Gaussian-derived random z-value, which is used with sigma to calculate a distance moved in the 2D-plane (thus, the use of 4Dt for average sigma). Collisions were not considered, i.e., there was no physical exclusion of overlapping molecular positions. This was an O(n) calculation.

Update Binding

If a ligand was considered present, the first routine cycles through each IgE data structure looking at each IgE Fab “arm” for the presence of bound ligand (code line 833 of S4_File); if not bound, a random number is generated and compared to the probability that within the time-step binding should occur (this is a Markov-chain calculation in that each binding reaction has the chance of occurring with a

probability determined by chemical reaction time constants. The probability of ligand binding within a given time-step interval is:

Probability of binding = 1- exp(-[ligand]*valency*K_f*timestep) (2)

Probability of dissociation = 1 - exp(-K_r*timestep) (3)

Where [ligand] is the concentration of ligand (M), valency is the number of binding sites per ligand, K_f is the forward rate binding constant ($M^{-1}sec^{-1}$), K_r is the dissociation constant (sec^{-1}, equation 3), and timestep, the fixed time interval for the entire simulation (sec). If the value of the obtained random floating point number for this specific event was less than the probability of binding, then binding was deemed to have occurred. This was an O(n) calculation. As noted previously, the time-step interval was chosen to accommodate a reasonable range of concentrations around the optimum for crosslinking (1/2K_a) [4, 5], values consistent with precision of the 'float' variables, the random number generator precision and more importantly, consistent with simulation producing results that matched analytical predictions for diffusion and crosslinking curves. If the ligand-binding site "arm" already had ligand bound, then a similar calculation was made for ligand dissociation (equation (3)). For each IgE, two such assessments were made for each potential ligand binding site.

Determine intersection between IgE/FceRI positions

One computational step was an $O(n^2)$ calculation, the examination of IgE/receptor interaction distance. Edge effects also needed to be accounted for as motion at the

edges of the planar space wrapped to the opposite side, so IgE/receptors could be in a cluster whose members were on "opposite" sides of the plane (left-to-right or top-to-bottom, or both). For actual basophils, differences in receptor density range over 100-fold. As an $O(n^2)$ calculation, this would increase simulation times 10000-fold. To reduce the computational burden, once receptor densities exceeded 50,000, the cell "size" was reduced. Therefore a 10 fold increase in receptor density was achieved by a 10 fold reduction in surface area (sqrt(10) in radius) without increasing the simulation time by a further 100-fold.

For the purposes of the simulation, a fixed distance threshold was required to consider the option of crosslinking. This distance was calculated from the physical characteristics of the IgE and the crosslinker [2, 4]. The intersectional distance was calculated for its relevance to the size of a small bivalent molecule as the ligand.

Crosslinking

For a model of ligand-induced crosslinking, crosslinking constants were derived from previous studies [3]. A detailed model of crosslinking has been explored and the while the rotational orientation of each IgE molecule would normally need consideration, as would other considerations of geometry, the rate of crosslinking can be reduced to a single forward and reverse rate. This single pair of rate constants were assumed to be adequate for this simulation [3] and for the simulation, the crosslinking constant was a constant and re-cast as a probability of linking within the time-step of the simulation; a value of 0.02. This crosslinking

constant is derived from a number of geometric considerations, the encounter complex ligand concentration and forward binding constant. For example, for a 3.5 nm bivalent ligand, the fraction of encounter complex geometries (see [2], radius of gyration = 6.75 nm (includes R_G), ligand pocket depth of 1.25 nm, hapten length = 3.5 nm) for which the ligand can reach from one binding site to a second site (vs. all possible encounters), in 3 dimensions, is approximately 2-4%. With the local concentration of ligand and forward binding rate (used for the bivalent ligand simulations), the probability of completing the crosslink is near 1.0 for the timestep used, resulting in an overall probability for a random encounter of approximately 0.02-0.04. It is a number with considerable possible range depending on the ligands and ligand geometry. Since the encounter complex requires a minimum threshold center-to-center distance to consider whether crosslinking has a chance of occurring, this distance is the value for the threshold of 13.5 nm used throughout the basic simulation. A crosslinking step could involve interactions of one bound site to one or two possible unbound sites or two bound sites linking to one or two single possible bound sites. The adjustment of the crosslinking reaction probabilities for these cases followed the scheme outlined in previous studies of bivalent interactions [3] (code lines 1331 or 1539 of S4_File). If the crosslinking step involved a bound site to two possible unbound sites, a Boolean random number generator was used to chose the site being bound and crosslinked if binding was determined to be successful. Parenthetically, the model could accommodate cyclic binding but previous experimental studies suggested that cyclic binding was minimal for simple bivalent haptens and this probability was set to zero. If a bound

site was found to become unbound during a time-step (see above) and the bound site was also involved in a crosslink, the crosslink was dissolved.

Linked list data structures

Linked list data structures were used to keep track of statistics on the simulation as well as manage the book-keeping for the simulation. The linked-list also allowed statistics on spontaneous aggregate size and aggregate lifetimes in the simulations where spontaneous aggregation was followed.

Assessing aggregate persistence

The linked lists allowed a measurement of aggregate persistence times by deriving from the linked lists whether a particular IgE was involved in an aggregate at each time cycle (code line 3742 of File_S4). The linked list for each time cycle was saved and later analysis worked through the lists for each IgE for the duration of the simulation. S2 Fig illustrates how data were accumulated.

Table 1: Constants used (see references [4, 6]):

Ligand binding forward rate	$5x10^{7}$	$M^{-1}sec^{-1}$
Ligand dissociation rate	10	sec^{-1}
Translational diffusion, D	$2x10^{-10}$	$cm^{2}sec^{-1}$
Crosslinking probability	0.02	(per encounter)
Threshold for proximity	13.5	nm
Radius of gyration, IgE	6.5	nm
Timestep	50	microseconds

Simulation Results

There were two areas explored with the simulation. The primary goal was to assess the dependence of cluster size and cluster association time with FceRI density. The secondary goal was to characterize a weak linear aggregator as a comparison to a ligand that is known to induce secretion.

Spontaneous Aggregation

S3_Fig summarizes the results for cluster size, without regard to duration of the clusters.

The fraction of receptors involved in clusters/aggregates increases to ≈13% (without consideration for size or persistence) of the total receptors at densities > 500,000 FceRI per cell (S3A fig) for a separation distance (center-to-center) of 13.5 nm or 20% for a separation distance of 20.0 nm. While the majority of clusters are dimeric, for 500,000 total receptors per cell, the cluster size distribution reaches as high as 7. These will be the clusters that persist for longer times since it takes some time for these non-associated clusters to reduce their size to monomers, with receptors both entering and exiting the cluster. The two distances 13.5 and 20 nm were obtained from two considerations. In the first case, this distance represents the center-to-center distance for the two IgE antibodies to nearly make contact at their radius of gyration. In some arm orientations, the distance between two ligand binding sites is just sufficient to accommodate a linkage with a short bivalent

crosslinker such as BPO2 (8-carbon backbone, = approx.. 3.5-4 nm). The second value, 20 nm was an estimate of FceRI density discovered by McConnell to occur in regions of the RBL cell that were not crosslinked but induced activation signals by the apparent virtue of their proximity [7]. The packing distances in his experimental RBL cell system suggested a center-to-center distance of 20 nm.

S4_Fig shows the persistence distribution for 2 scenarios; distance separations of either 13.5 or 20.0 nm. It is apparent that at high densities, persistence times on the order of 10 msec exist and if the density of receptor is in the hundreds of thousands, several hundred such persistent clusters exist. But what is not apparent in the figure as shown is that there were approximately 3.5 million associations that lasted less than 250 microseconds. Only 2% of the associations persist for longer than 2 msec.

Ligand-Induced Aggregation

To provide context for these spontaneous aggregation simulations, simulations of ligand-induced crosslinking were made. To model the weakest of ligands that continue to induce histamine release, the affinity constants for ligand binding matched the determined affinity constants for BPO_2 (K_a = 5x10^6, k_f = 5x10^7 $M^{-1}sec^{-1}$, k_r = 10 sec^{-1}). This is a fast off-rate but experimentally, a BPO_2 with this affinity induces secretion [4, 8].

A first test of the ligand binding simulation was to determine if a "concentration-dependence" for the crosslinking curve obeyed the known property that the peak of

crosslinking occurs at $1/2K_a$ [5]. This was found to be true for the simulation (see S5_Fig).

S6 Fig shows the crosslink duration distribution for this simple bivalent reaction. The first point to note that is not apparent in the figure is that short duration aggregates account for the majority of the aggregates but this is because spontaneous aggregates continue to occur. That said, while there may be a cumulative 3.5 million "aggregates" (within the time frame of the simulation) that persist for less than 250 microseconds in the non-crosslinked simulation, this number is less than 1 million when a crosslinker is present. This results from actual crosslinks ultimately dominating the development of aggregates (i.e., true crosslinks). Many of these still have short persistence times but are generally considerably longer than spontaneous aggregates. Nevertheless the average time is skewed towards the early state of the simulation and to the short persistence of the still considerable number of spontaneous aggregates.

S6_Fig shows, however, that there are many long-lived events. Compare this distribution with the short-tailed distribution for the persistence of spontaneous aggregates. While a few spontaneous aggregates persist for 10 msecs, even a simple low affinity bivalent crosslinker has many more aggregates that persist for >10 milliseconds when the density is the same as for the spontaneous aggregate simulation. For panel A, the average persistence was 0.67 msecs while for the reaction with crosslinking, it was approximately 2 msecs. But excluding the bias

introduced by the continuing presence of many spontaneous aggregates (i.e., only include in the average aggregates persisting for longer than 10 msecs because few spontaneous aggregates persist longer than 10 msecs), the aggregate persistence for a weak crosslinker is approximately 60 msecs. At the same density of 270,000/cell, the curves in panel A are obviously very different. However, if the receptor is decreased to densities known to still be sufficient to induce histamine release but far from saturated, the average persistence time remains similar to higher densities (in fact, the average even without excluding persistence times <10 msec remains because spontaneous aggregates nearly disappear (see S3_Fig) leaving only aggregates that were formed by the crosslinker, giving a more accurate picture of the average persistence). By causal inspection, the results for spontaneous aggregates at a density of 270,000/cell and crosslinked aggregates at a density of 13500/cell look similar, but the persistence for crosslinked aggregates remains much longer and 19% (≈1200 aggregates) of total aggregates persist for >10 msec. Likewise, when the concentration of crosslinker is reduced to generate a distribution more similar to spontaneous aggregates, while maintaining density at 270,000/cell, the absolute numbers below 20 msecs duration are less but there is a long "tail" of persistent aggregates (≈6100 aggregates in the "tail", with an average persistence of 50 msecs.). Increasing the dissociation constant for this reaction by 10-fold (k_r = 100 sec^{-1}) reduces the number of aggregates that persist longer than 10 msec by 45-fold (≈ 2%) (S6C Fig) and most of these are between 10-15 msec in duration.

References for Appendix 1 supporting information

1. Press, W. H., Flannery, B. P., Teukolsky, S. A., Vetterling, W. T. (1988) Numerical Recipes in C. Cambridge University Press, Cambridge.
2. Dembo, M. and Goldstein, B. (1978) Theory of equilibrium binding of symmetric bivalent haptens to cell surface antibody: application to histamine release from basophils. J. Immunol. 121, 345-353.
3. Dembo, M., Goldstein, B., Sobotka, A. K., Lichtenstein, L. M. (1979) Histamine release due to bivalent penicilloyl haptens: the relation of activation and desensitization of basophils to dynamic aspects of ligand binding to cell surface antibody. J Immunol 122, 518-28.
4. Dembo, M., Goldstein, B., Sobotka, A. K., Lichtenstein, L. M. (1978) Histamine release due to bivalent penicilloyl haptens: Control by the number of cross-linked IgE antibodies on the basophil plasma membrane. J Immunol 121, 354-8.
5. MacGlashan, D. W., Jr., Dembo, M., Goldstein, B. (1985) Test of a theory relating to the cross-linking of IgE antibody on the surface of human basophils. J Immunol 135, 4129-34.
6. Dembo, M., Goldstein, B., Sobotka, A. K., Lichtenstein, L. M. (1979) Degranulation of human basophils: quantitative analysis of histamine release and desensitization, due to a bivalent penicilloyl hapten. J Immunol 123, 1864-72.
7. Weis, R. M., Balakrishnan, K., Smith, B. A., McConnell, H. M. (1982) Stimulation of fluorescence in a small contact region between rat basophil

leukemia cells and planar lipid membrane targets by coherent evanescent radiation. J Biol Chem 257, 6440-5.

8. MacGlashan, D. W., Jr., Peters, S. P., Warner, J., Lichtenstein, L. M. (1986) Characteristics of human basophil sulfidopeptide leukotriene release: releasability defined as the ability of the basophil to respond to dimeric cross-links. J Immunol 136, 2231-9.
