# Supplementary figures and images for: FceRI density and spontaneous secretion from human basophils
Source: PLoS One. 2017 Jul 3;12(7):e0179734. doi: 10.1371/journal.pone.0179734 (PMC5495218; doi:10.1371/journal.pone.0179734)

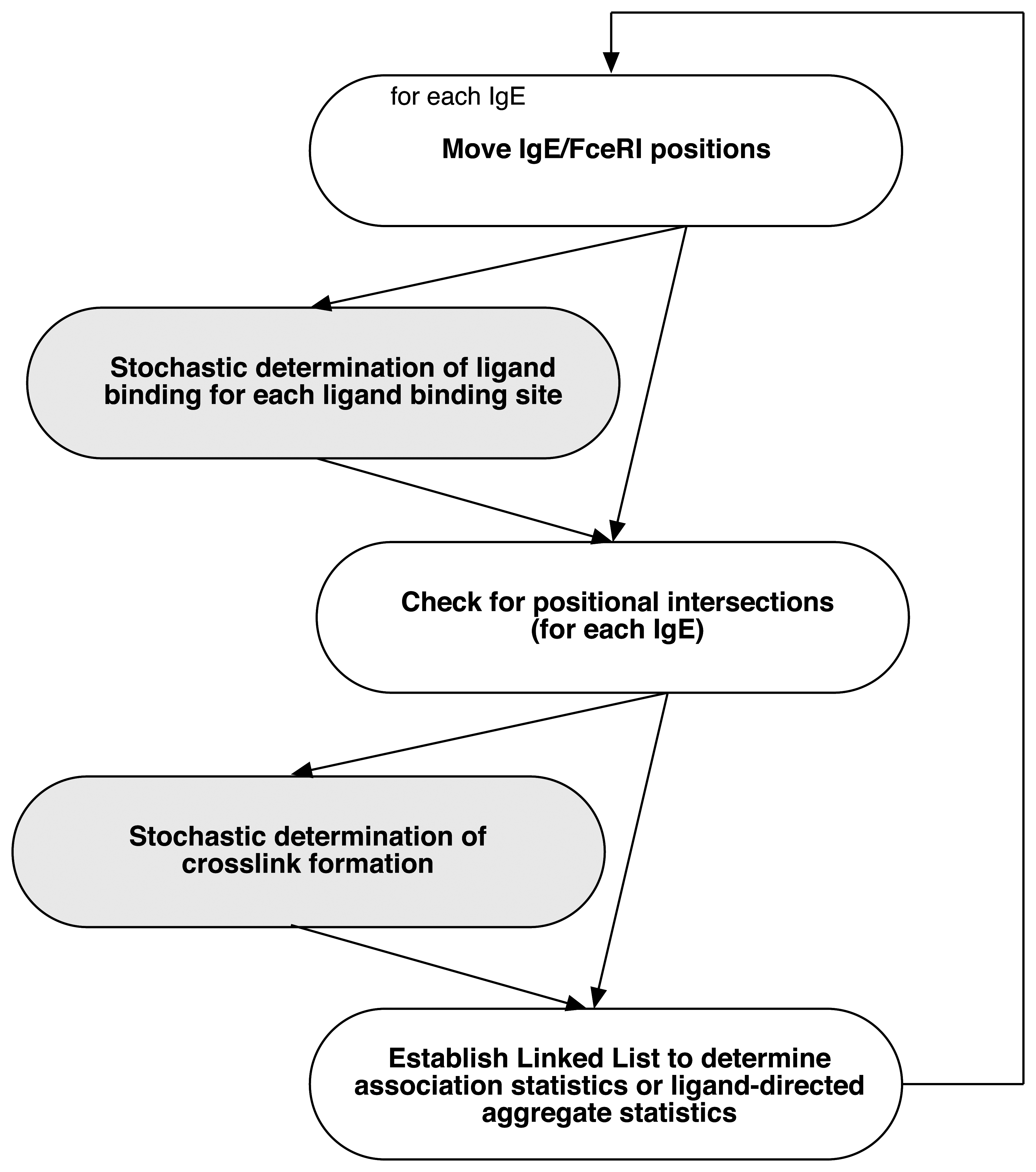

Supplement: S1 Fig — (TIF) [file pone.0179734.s005.tif]

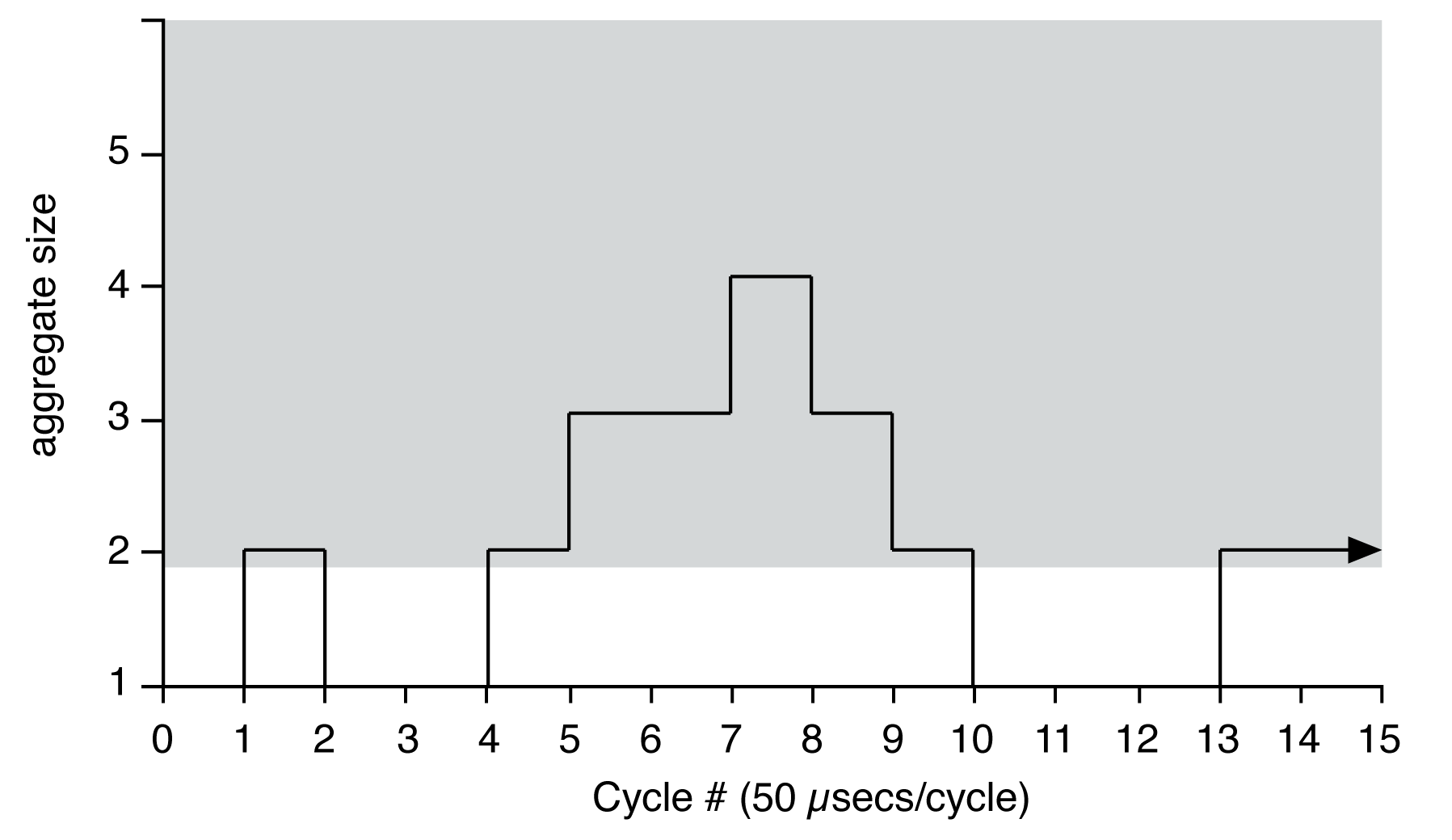

Supplement: S2 Fig — Each IgE molecule was tracked for its association with an aggregate and marked for size of the aggregate at each cycle. A record for all IgEs at each cycle was stored and later scanned for each IgE and its status with respect to aggregate size. If it was marked as in an aggregate of size ≥2 (the gray zone in Fig 3), then a counter was started. Once returning to size 1, the counter was stopped and the length of time tallied. The figures shows one particular IgE for a period of only 15 cycles (750 µsec) and it would count 2 events (1 only 50 µsec in length and one 300 µsec in length and passing briefly through an aggregate of size 4) and the start of a third event cut short in this plot. (TIF) [file pone.0179734.s006.tif]

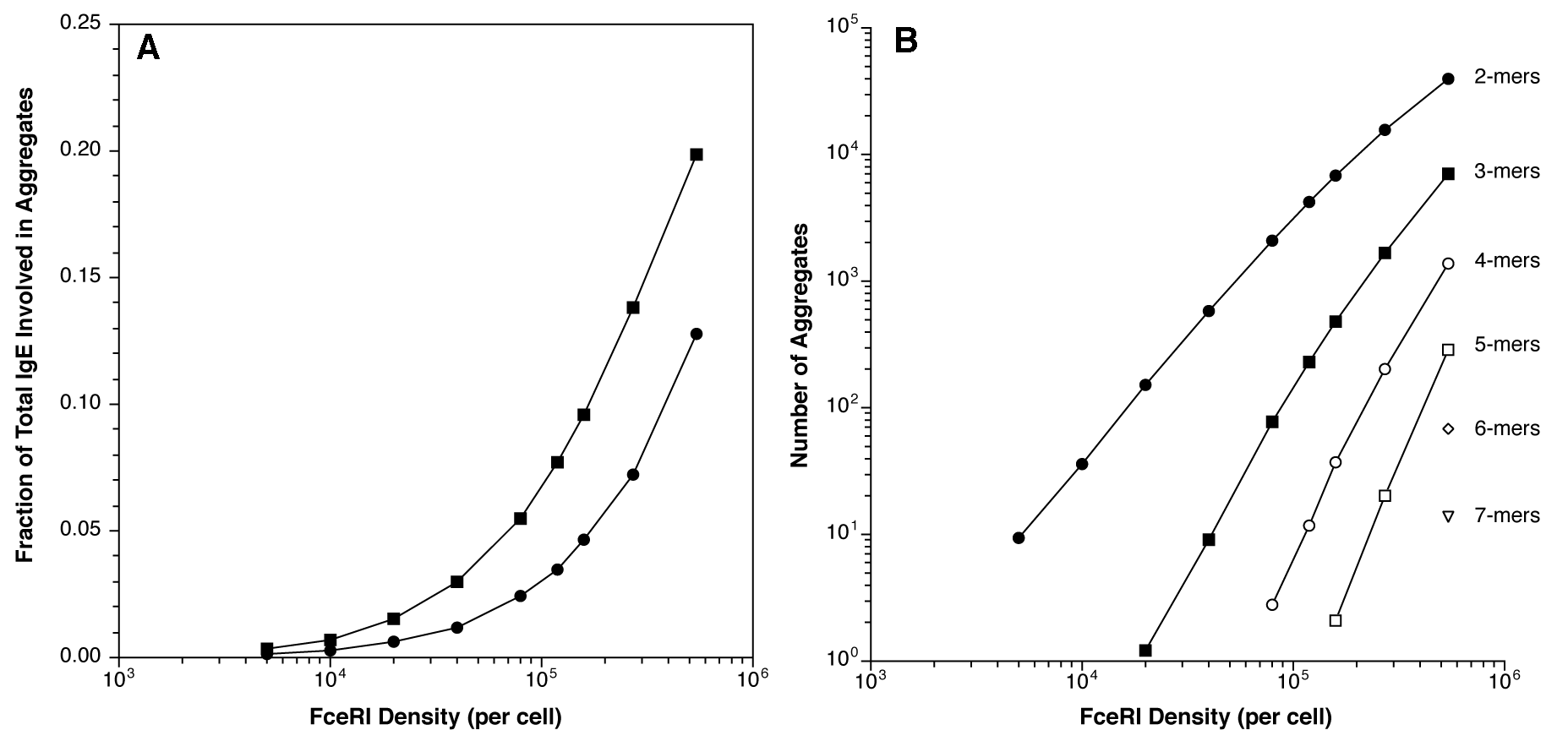

Supplement: S3 Fig — Panel A; results of simulation for a 13.5 (filled circles) and 20.0 (filled squares) nm center-to-center separation distance being counted as an aggregate without regard to size or persistence at a given moment. Panel B; relationship between the size of the spontaneous aggregate, the number of aggregates and density of FceRI. The numbers on the ordinate reflect the total number of aggregates (to calculate the total number of IgE/receptor involved, multiply by the aggregate size (e.g., a pentamer aggregate = 5 total receptors involved). (TIF) [file pone.0179734.s007.tif]

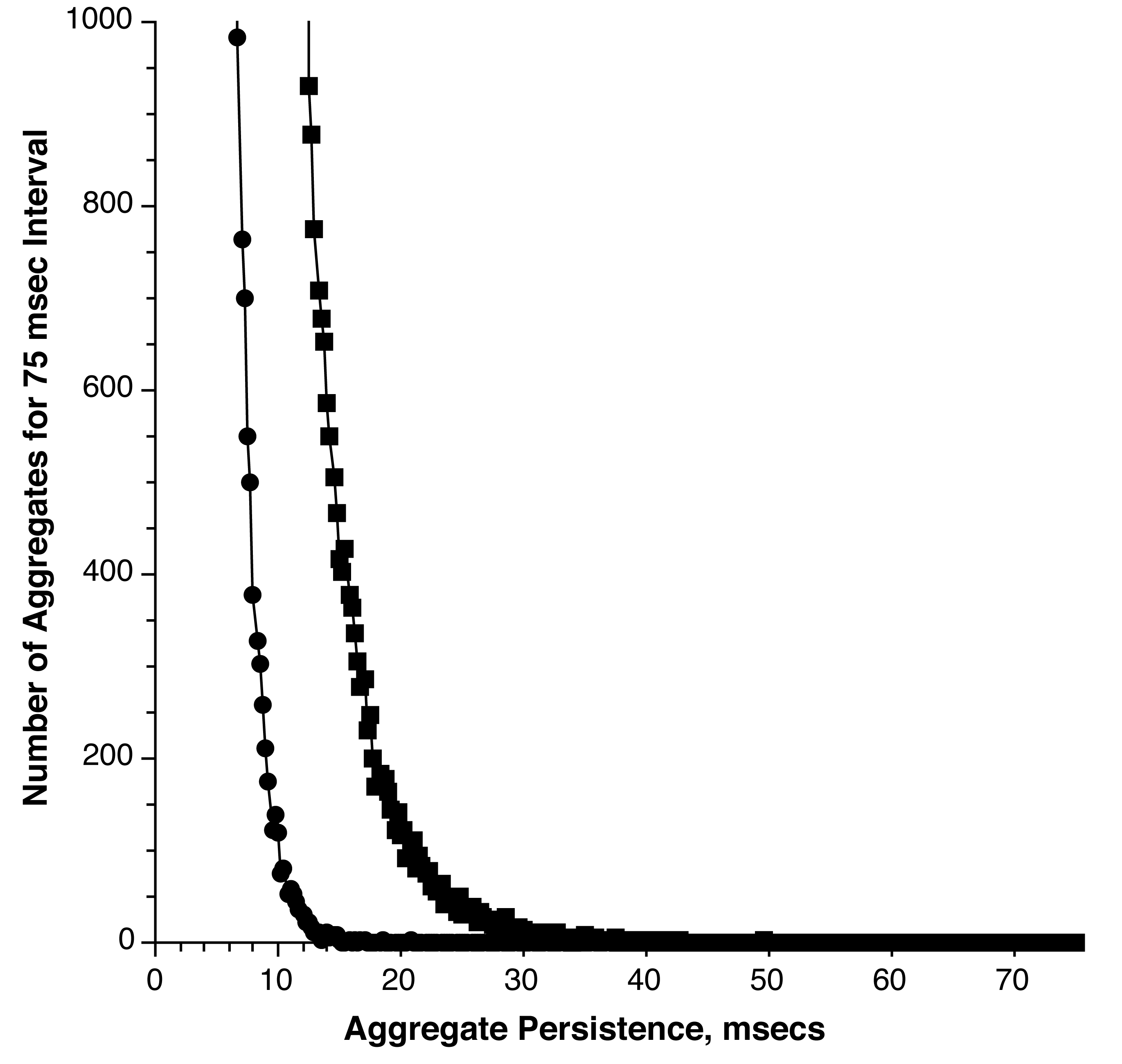

Supplement: S4 Fig — Persistence for clusters/aggregates of any size larger than monomers for two center-to-center separation distances (circle = 13.5 nm, square = 20.0 nm). The count is for the number of such clusters for the first 75 msec of the simulation at a total receptor density of 270,000. (TIF) [file pone.0179734.s008.tif]

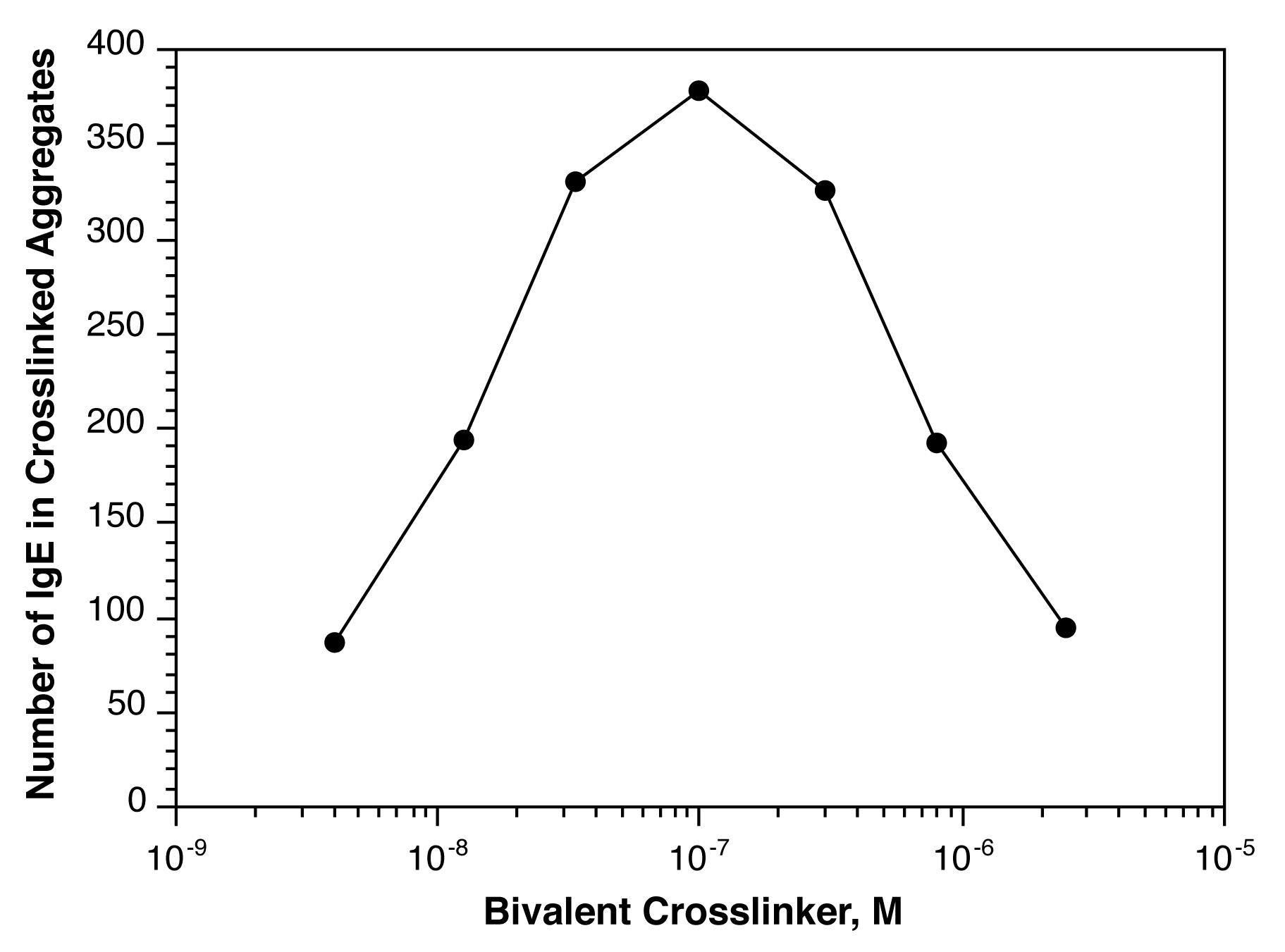

Supplement: S5 Fig — A single-site affinity of 5x10^6^ M-1 was used for the simulation. (TIF) [file pone.0179734.s009.tif]

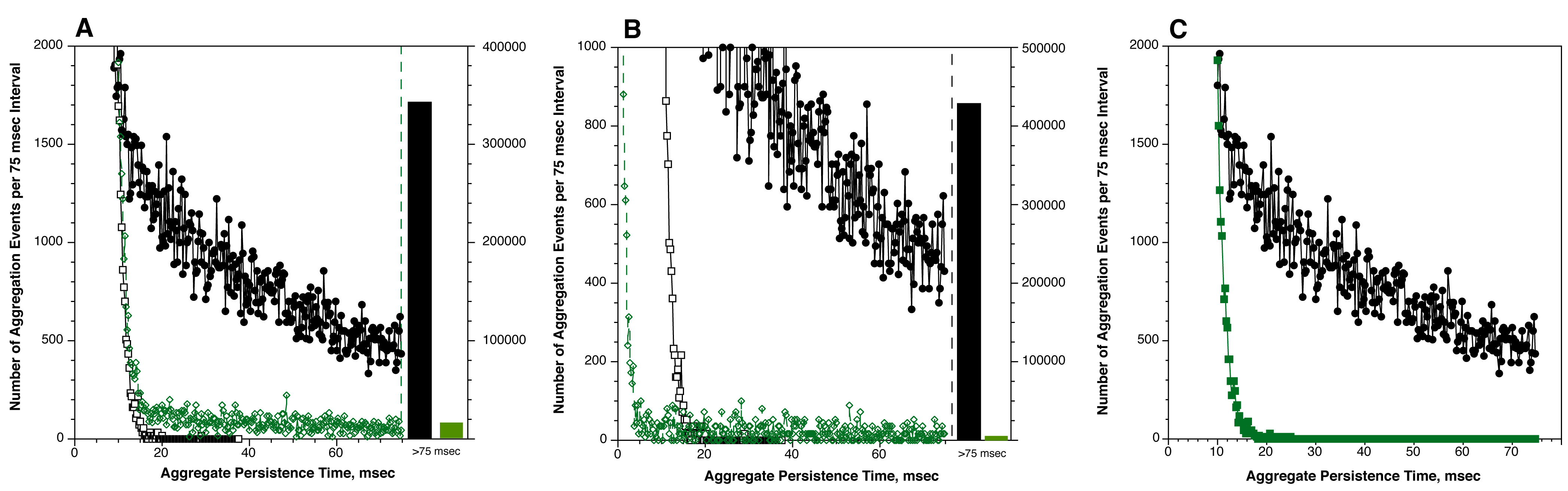

Supplement: S6 Fig — Panel A, 3 simulations, all at 270,000 receptors/cell, spontaneous aggregates (open squares), an optimal concentration (10−7 M) of crosslinker (black circles), or suboptimal crosslinker (2.5x10^-10^ M) (green open diamonds). The histograms on the right edge of the plot show the remaining number of aggregates at times > 75 msecs. Panel B, 3 simulations, at two different densities of receptor; spontaneous aggregates at 270,000 receptors/cell (open squares), an optimal concentration (10−7 M) of crosslinker at 270,000 receptors/cell (black circles), or optimal crosslinker (10−7 M) at 13,500 receptors/cell (green open circles). The histograms on the right edge of the plot show the remaining number of aggregates at times > 75 msecs. Panel C; 2 simulations, kr (dissociation constant) = 10 (, as in panel A) or = 100 (green squares) both at 270,000 receptors/cell. (TIF) [file pone.0179734.s010.tif]
